# Supplementary material for: Hybrid Models and Biological Model Reduction with PyDSTool
Source: PLoS Comput Biol. 2012 Aug 9;8(8):e1002628. doi: 10.1371/journal.pcbi.1002628 (PMC3415397; doi:10.1371/journal.pcbi.1002628)
Supplement: Text S4 — Complete source code for the PyDSTool package (version 0.88.120504). Includes API documentation and help files linking to web pages. This file is identical to the current public release on Sourceforge.net. (ZIP) [file pcbi.1002628.s004.zip › PyDSTool/html/PyDSTool.MProject.GeneratorInterface-class.html]

xml version="1.0" encoding="ascii"?


PyDSTool.MProject.GeneratorInterface


| Home | Trees | Indices | Help | PyDSTool |
| --- | --- | --- | --- | --- |

Package PyDSTool ::
Module MProject ::
Class GeneratorInterface

[hide private]

[frames] | no frames]

# Class GeneratorInterface

source code

```
 object --+    
          |    
dsInterface --+
              |
             GeneratorInterface
```

---

Wrapper for Generator (for non-hybrid models) that shares similar API
with ModelInterface for use in HybridModel objects.


|  |  |
| --- | --- |
| Instance Methods | [hide private] |

|  |  |
| --- | --- |
| \_\_init\_\_(self, model, FScompatibleNames=None, FScompatibleNamesInv=None)  model argument must be a Generator only | source code |

|  |  |
| --- | --- |
| get(self, key, ics=None, t0=0) | source code |

|  |  |
| --- | --- |
| set(self, key, value, ics=None, t0=0) | source code |

|  |  |
| --- | --- |
| Rhs(self, t, xdict, pdict)  Direct access to a generator's Rhs function. | source code |

|  |  |
| --- | --- |
| Jacobian(self, t, xdict, pdict, idict=None)  Direct access to a generator's Jacobian function (if defined). | source code |

|  |  |
| --- | --- |
| JacobianP(self, t, xdict, pdict)  Direct access to a generator's JacobianP function (if defined). | source code |

|  |  |
| --- | --- |
| MassMatrix(self, t, xdict, pdict)  Direct access to a generator's MassMatrix function (if defined). | source code |

|  |  |
| --- | --- |
| AuxVars(self, t, xdict, pdict)  Direct access to a generator's auxiliary variables definition (if defined). | source code |

**Inherited from `dsInterface`**:
`get_test_traj`,
`query`

**Inherited from `object`**:
`__delattr__`,
`__getattribute__`,
`__hash__`,
`__new__`,
`__reduce__`,
`__reduce_ex__`,
`__repr__`,
`__setattr__`,
`__str__`


|  |  |
| --- | --- |
| Class Variables | [hide private] |

**Inherited from `dsInterface`** (private):
`_querykeys`,
`_setkeys`


|  |  |
| --- | --- |
| Properties | [hide private] |

**Inherited from `object`**:
`__class__`


|  |  |
| --- | --- |
| Method Details | [hide private] |

|  |  |
| --- | --- |
| \_\_init\_\_(self, model, FScompatibleNames=None, FScompatibleNamesInv=None)  *(Constructor)* | source code |

model argument must be a Generator only

Overrides: object.\_\_init\_\_

  


| Home | Trees | Indices | Help | PyDSTool |
| --- | --- | --- | --- | --- |

|  |  |
| --- | --- |
| Generated by Epydoc 3.0.1 on Fri May 4 15:24:06 2012 | http://epydoc.sourceforge.net |
